# A Smartphone Intervention for People With Serious Mental Illness: Fully Remote Randomized Controlled Trial of CORE

**DOI:** 10.2196/29201

**Published:** 2021-11-12

**Authors:** Dror Ben-Zeev, Ayesha Chander, Justin Tauscher, Benjamin Buck, Subigya Nepal, Andrew Campbell, Guy Doron

**Affiliations:** 1 Behavioral Research in Technology and Engineering Center Department of Psychiatry and Behavioral Sciences University of Washington Seattle, WA United States; 2 Department of Computer Science Dartmouth College Hanover, NH United States; 3 School of Psychology Interdisciplinary Center Herzliya Israel

**Keywords:** mobile health, schizophrenia, bipolar disorder, depression, mobile phone

## Abstract

**Background:**

People with serious mental illness (SMI) have significant unmet mental health needs. Development and testing of digital interventions that can alleviate the suffering of people with SMI is a public health priority.

**Objective:**

The aim of this study is to conduct a fully remote randomized waitlist-controlled trial of CORE, a smartphone intervention that comprises daily exercises designed to promote reassessment of dysfunctional beliefs in multiple domains.

**Methods:**

Individuals were recruited via the web using Google and Facebook advertisements. Enrolled participants were randomized into either active intervention or waitlist control groups. Participants completed the Beck Depression Inventory-Second Edition (BDI-II), Generalized Anxiety Disorder-7 (GAD-7), Hamilton Program for Schizophrenia Voices, Green Paranoid Thought Scale, Recovery Assessment Scale (RAS), Rosenberg Self-Esteem Scale (RSES), Friendship Scale, and Sheehan Disability Scale (SDS) at baseline (T1), 30-day (T2), and 60-day (T3) assessment points. Participants in the active group used CORE from T1 to T2, and participants in the waitlist group used CORE from T2 to T3. Both groups completed usability and accessibility measures after they concluded their intervention periods.

**Results:**

Overall, 315 individuals from 45 states participated in this study. The sample comprised individuals with self-reported bipolar disorder (111/315, 35.2%), major depressive disorder (136/315, 43.2%), and schizophrenia or schizoaffective disorder (68/315, 21.6%) who displayed moderate to severe symptoms and disability levels at baseline. Participants rated CORE as highly usable and acceptable. Intent-to-treat analyses showed significant treatment×time interactions for the BDI-II (*F*_1,313_=13.38; *P<*.001), GAD-7 (*F*_1,313_=5.87; *P*=.01), RAS (*F*_1,313_=23.42; *P<*.001), RSES (*F*_1,313_=19.28; *P<*.001), and SDS (*F*_1,313_=10.73; *P*=.001). Large effects were observed for the BDI-II (*d=*0.58), RAS (*d=*0.61), and RSES (*d=*0.64); a moderate effect size was observed for the SDS (*d=*0.44), and a small effect size was observed for the GAD-7 (*d=*0.20). Similar changes in outcome measures were later observed in the waitlist control group participants following crossover after they received CORE (T2 to T3). Approximately 41.5% (64/154) of participants in the active group and 60.2% (97/161) of participants in the waitlist group were retained at T2, and 33.1% (51/154) of participants in the active group and 40.3% (65/161) of participants in the waitlist group were retained at T3.

**Conclusions:**

We successfully recruited, screened, randomized, treated, and assessed a geographically dispersed sample of participants with SMI entirely via the web, demonstrating that fully remote clinical trials are feasible in this population; however, study retention remains challenging. CORE showed promise as a usable, acceptable, and effective tool for reducing the severity of psychiatric symptoms and disability while improving recovery and self-esteem. Rapid adoption and real-world dissemination of evidence-based mobile health interventions such as CORE are needed if we are to shorten the science-to-service gap and address the significant unmet mental health needs of people with SMI during the COVID-19 pandemic and beyond.

**Trial Registration:**

ClinicalTrials.gov NCT04068467; https://clinicaltrials.gov/ct2/show/NCT04068467

## Introduction

### Background

People with serious mental illnesses (SMIs), including schizophrenia-spectrum disorders, bipolar disorder, and severe and persistent depression, experience significant psychiatric symptoms such as hallucinations, delusions, and severe mood episodes. SMI is often accompanied by functional and psychosocial impairments, housing and employment challenges, and poverty [1-3]. Treatment of people with SMI typically takes place in publicly funded clinics and community mental health centers that are chronically underresourced, understaffed, and overextended [4,5]. These public sector agencies are rarely able to meet the demand for services [6]. Barriers in the capacity to provide high-quality care on the provider’s side may be compounded by hesitancy around treatment seeking on the patient’s side [7]. People with SMI are often exposed to pervasive societal stigma about their conditions and therefore can be reluctant to openly seek services at a clinic if it risks them being labeled *mentally ill* [8,9]. The consequence of these intersecting challenges is a vicious cycle in which those who are most impaired receive the least amount of support, thus deteriorating even more over time.

People with SMI are not dramatically different from the general population in their use of mobile technologies [10,11]. Owing to subsidy programs that offer people with disabilities access to mobile phones and data plans (eg, Federal Communications Commission Lifeline Program) and the dropping prices of mobile phones worldwide, people with SMI now have unprecedented opportunities to access information, stay connected to others, and potentially receive services remotely via personal mobile devices [12]. Survey studies conducted in recent years have shown that approximately two-thirds of people with SMI in the United States already own smartphones and that most are interested in leveraging them to support their health care [13-16]. Academic research groups and commercial companies are responding to the opportunity and have begun to develop and test a range of smartphone-supported digital health technologies for people with SMI, including illness self-management apps, virtual peer support platforms, and sensor-enabled remote patient monitoring systems [17-19].

Conducting the research necessary to demonstrate the safety and clinical utility of novel smartphone interventions for people with SMI is challenging and costly. Recruitment of participants for clinical trials is the most salient cause of study delays and a major obstacle in expeditiously moving novel and potentially helpful interventions to real-world practice [20]. Traditional participant recruitment strategies are dependent on collaborative partnerships with study sites and clinician referrals [21]. This creates potential biases in clinical trial samples, as the enrolled individuals already need to be known by health care providers and be actively receiving care to be offered the opportunity to engage in research. Therefore, the participant samples used in standard clinical trials may be particularly unrepresentative of hard-to-reach populations, such as people with SMI, who are known to disengage from clinic-based services for extended periods [22,23].

To overcome these obstacles, a growing number of studies have used web-based participant recruitment strategies, including social media advertisements, virtual outreach through web-based interest groups, search engine advertisements, and various other website campaigns. Across clinical populations, these efforts have yielded impressive results in terms of cost-effectiveness, time efficiency, and reach [24]. In clinical trials where the experimental intervention is digital and behavioral in nature (ie, does not require direct contact between a patient and a provider) and the comparator condition does not require in-person contact with the research team (eg, no intervention control, waitlist control, or digital attention–control alternative), recruitment, treatment, and assessment of outcomes can all be conducted fully remotely and potentially on a single device [25-30]. To our knowledge, fully remote randomized controlled trials have not been conducted with people with SMI [24].

### Objective

Here, we report the first fully remote randomized controlled clinical trial of a mobile health (mHealth) intervention for people with SMI. The first objective of this study is to evaluate whether individuals with SMI can be successfully recruited, assessed, and engaged in a digital intervention in a fully remote clinical trial. The second objective of the study is to evaluate the clinical effectiveness of the CORE intervention, a smartphone app designed to challenge dysfunctional thoughts that underlie common symptoms of SMI, self-stigmatizing attitudes, and maladaptive beliefs that impede treatment seeking and recovery.

## Methods

### Study Design

This study involved a fully remote randomized controlled crossover waitlist trial design. All participants were given the opportunity to receive the study intervention. Following enrollment, participants were randomized to either receiving the CORE intervention (indicated as *1*) or being in the waitlist control group (indicated as *0*) in blocks of 4. The group allocation for each block was selected randomly from a list of 6 possible sequences (ie, 1100, 1010, 1001, 0110, 0101, and 0011). In the active intervention group, participants completed the baseline assessment and were then immediately given access to the CORE app for 30 days of use. After a month, they concluded the intervention, uninstalled the app, and completed a second assessment. After an additional month (at 60 days), they completed a third assessment to measure the stability of symptom change post intervention. In the control arm, participants waited 30 days to receive the CORE app. After a month, they completed a second assessment and were provided access to the CORE app. After an additional month (at 60 days), they completed a third assessment to measure within-subject changes.

### Procedures

All study procedures were reviewed and approved by the institutional review board of the University of Washington (ID number 00006898). We put a detailed and institutional review board–approved plan in place to respond to cases of increased risk. Web-based recruitment was conducted through advertisements on Google and Facebook. Google advertisements are presented depending on the user’s search terms. For this study, we selected a range of terms associated with severe mental illness (eg, *schizophrenia, bipolar*, *seeing things,* and *am I crazy*) and related keywords generated by the Google *broad match* algorithm. Facebook advertisements use a similar methodology to target individual interests. For this study, we targeted interests such as mental health and schizophrenia. Individuals who clicked on the advertisements were directed to the study website. The study website provided written and video descriptions of the project, a downloadable version of the study consent form, and an option to complete a screening questionnaire via a *see if I am eligible* button. If eligible, participants were again presented with the consent form and were required to answer questions demonstrating their understanding of the study details. If they answered these questions correctly within 3 attempts, they could proceed to the baseline assessments. Participants were excluded if they were unable to complete this step successfully, as they were deemed unable to provide informed consent. Participants completed a battery of self-report questionnaires on the web at baseline. If participants’ responses to questionnaires indicated the presence of suicidal ideation, they were immediately provided with resources for emergency support. Upon completion of baseline questionnaires, participants were randomized to either an active intervention or waitlist control group. Those randomized to the active intervention arm were given instructions on how to download and install the CORE mobile app.

At the start of the study period, staff contacted participants via email or SMS text messaging to welcome them to the study and remind them to complete the follow-up assessments at 30 and 60 days. After this welcome message, participants interacted with the study team only if they reached out to the study staff for technical support. When participants were due for an assessment, they were sent an automated SMS text message to alert them to complete the battery through a weblink. Participants were incentivized by being entered into lotteries to win US $500 and US $1000 after completing the 30- and 60-day assessments, respectively.

A member (JT) of our research team who was blinded to participant allocation completed all study analyses, and these analyses were determined beforehand. All outcome measures were preselected by the study team and examined in this study. The sample size was calculated to fit analytic requirements, and study recruitment was discontinued once the required sample size was met.

### Participants

Participants were eligible if they could speak English; were aged >18 years; lived in the United States; self-reported a diagnosis of schizophrenia or schizoaffective disorder, bipolar disorder, or major depressive disorder; and owned a smartphone with a data plan. They were excluded if they had previously participated in the study or were unavailable for 60 days of participation.

### Assessments

At all assessments, we administered measures of depression (Beck Depression Inventory–II [BDI-II] [31]), anxiety (Generalized Anxiety Disorder–7 [GAD-7] [32]), auditory hallucinations (Hamilton Program for Schizophrenia Voices Questionnaire [HPSVQ] [33]), paranoid thinking (Green Paranoid Thoughts Scale [GPTS] [34]), recovery (Recovery Assessment Scale [RAS] [35,36]), self-esteem (Rosenberg Self-Esteem Scale [RSES] [37]), social isolation (The Friendship Scale [38]), and functional disability (Sheehan Disability Scale [SDS] [39]). At baseline, we collected information from participants regarding demographics and technology use. After the intervention period, participants also completed a 26-item self-report usability and acceptability measure comprising adapted items from the System Usability Scale [40], Post Study System Usability Questionnaire [40], Technology Assessment Model Measurement Scales [41], and Usefulness, Satisfaction, and Ease questionnaire [42]. Participants were asked to rate their agreement with a series of statements about the intervention. A similar measure was used in previous studies conducted by our group [43].

### Intervention Description

The CORE intervention is a smartphone app that uses the GGtude platform, a system designed to increase the cognitive flexibility of individuals struggling with a range of mental health problems through brief daily training [44-47]. CORE was specifically designed to help counteract dysfunctional thoughts in multiple domains that are relevant to the subjective experience of having SMI. The intervention comprises daily brief game-like exercises designed to produce changes in the relative activation of adaptive and maladaptive beliefs about the self, others, and the world such that adaptive beliefs would be more easily retrieved than maladaptive ones.

**Figure 1:**
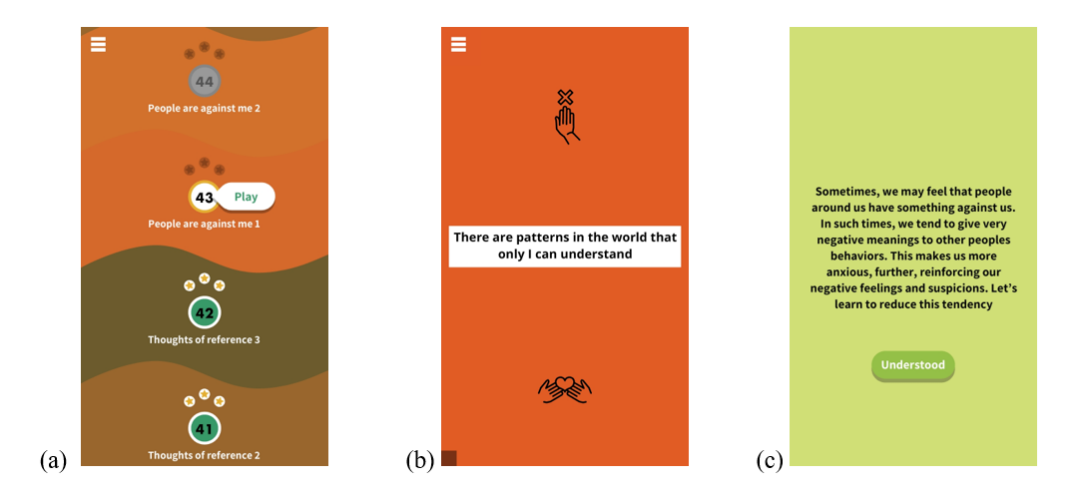
Screenshots of the (a) targeted beliefs menu, (b) an example of a maladaptive statement, and (c) a psychoeducational element.

CORE users were trained to respond to multiple statements in a sequence of modules that progress through the following domains: *self-talk, belief in change, self-stigma, self-care, self-worth, illness and identity, personal strength, social avoidance, feelings versus facts, catastrophization, thoughts of reference, paranoid ideation* (ie, *people are against me*), *treatment seeking,* and *recovery*. The modules begin with brief psychoeducation about the target domain and how maladaptive beliefs can hamper recovery. Thoughts appear as statements on the smartphone screen, and users are required to either endorse (ie, *drag* the statement down toward them on the touchscreen) or discard them (ie, *push* the statement upwards away from them). Users learn to embrace self-statements reflecting more nuanced adaptive thoughts (eg, belief in change, importance of self-care, alternative explanations to threat perceptions, and value of treatment seeking). The module content was organized into 53 levels, and participants were recommended to not complete more than 3 levels a day for a period of 30 days. Push notifications reminded users to complete their daily training. After each level, participants had the option of adding to their personal toolbox 1 of 3 positive statements that they most related to. Participants could access their toolbox at any time. Figure 1 depicts screenshots of the targeted beliefs menu, an example of a maladaptive statement, and a psychoeducational element.

### Data Analysis

Means, SDs, and frequencies were reported using descriptive statistics for all participant demographics, symptom measures, and acceptability and usability scale items. A series of *t* tests and chi-square tests were used to test for group differences in demographic variables and symptom measures at baseline (T1) before examining intervention effects at 30 days (T2) and 60 days (T3). Pre- and postscores for both groups were explored using a series of repeated-measures analysis of variance (ANOVA). The intervention effect between groups at T2 was initially tested using a 2×2 mixed-design repeated-measures ANOVA, controlling for participant diagnosis and baseline assessment scores. A 1×3 repeated measures ANOVA tested the performance of the active group across T1, T2, and T3, with post hoc Bonferroni pairwise comparisons used to explore outcomes when significant differences occurred. Finally, a series of 1×3 repeated measures ANOVAs tested the intervention effect after crossover (T2 and T3) for the waitlist control group participants to see if intervention effects were replicated. A series of equivalence tests were conducted using independent sample 2-tailed *t* tests to determine whether the magnitude of intervention effects differed for the active group participants between T1 and T2 as compared with the intervention effect for waitlist control participants between T2 and T3. Spaghetti plots were provided to visualize changes over time across symptom measures in both groups. To minimize bias during analysis, we used an intent-to-treat approach and implemented a multiple imputation strategy to replace missing values [48]. Multiple imputation is considered the most appropriate method of handling missing data for a study of this size, has been used successfully in repeated measure designs, and has been shown to have utility with levels of missing data greater than that observed in this study [49,50]. Sensitivity analyses using pattern-mixture models were used to establish data that were missing at random before imputation [51]. Our imputation model was specified using data from participants in each condition and included participant demographic variables (ie, diagnosis, gender, age, and race) and all baseline assessment scores.

## Results

### Recruitment and Enrollment

**Figure 2:**
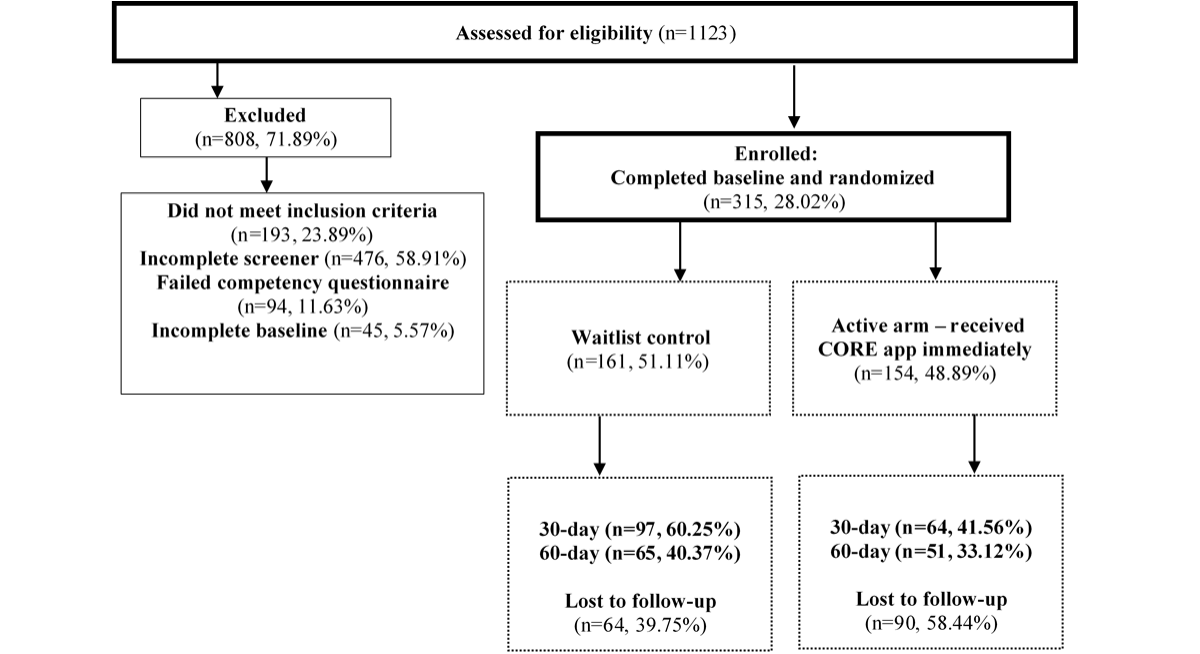
CONSORT (Consolidated Standards of Reporting Trials) diagram.

**Figure 3:**
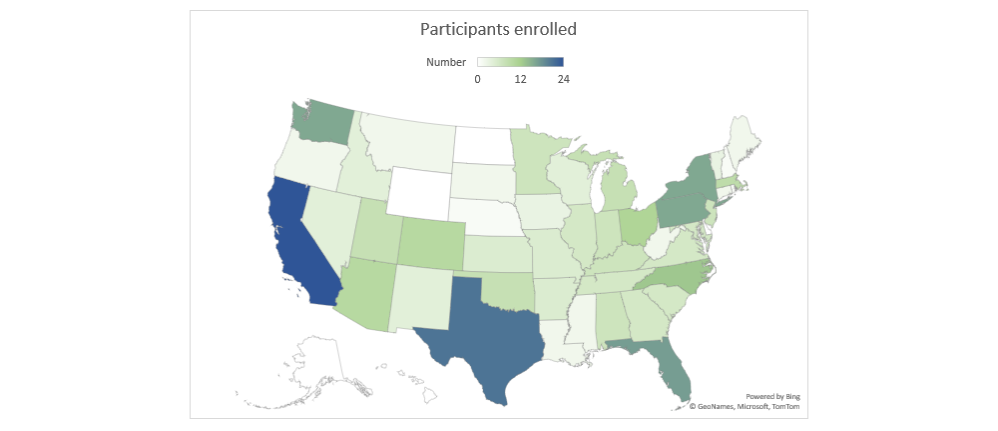
Map of participants enrolled based on location.

Web-based recruitment advertisements were placed between January 2020 and September 2020, using a total budget of US $2984.26. We recruited a total of 1123 people from these advertisements; of these 1123 people, 315 (28.04%) were randomized for the trial, and 808 (71.95%) were excluded. Of the 315 participants, 154 (48.9%) dropped out of the study before T2 and 45 (14.3%) dropped out between T2 and T3. Approximately 41.5% (64/154) of participants in the active group and 60.2% (97/161) of participants in the waitlist group were retained at T2, and 33.1% (51/154) of participants in the active group and 40.3% (65/161) of individuals in the waitlist group were retained at T3. Those who dropped out of the study were not found to differ significantly from those who were retained in terms of demographic characteristics or baseline scores on outcome measures (Figure 2). Most participants were recruited from Google advertisements (226/315, 71.7%). Overall, Google advertisements were viewed 557,700 times and were ultimately clicked on 5100 times. The states with the most clicks on the Google advertisement were California (540/5100, 10.59%), Texas (393/5100, 7.71%), Florida (295/5100, 5.78%), and New York (294/5100, 5.77%). The most successful Google advertisement keywords for increasing engagement from potential participants were *mental health*, *mental illness*, *depression*, *illuminati*, and *bipolar*. Most individuals (4734/5100, 92.82%) clicked on the advertisement through mobile phones, whereas few used desktop computers (256/5100, 5.02%) or tablets (110/5100, 2.16%). Participants that enrolled in the study were recruited from 45 states (Figure 3), with the most participants coming from California (24/315, 7.6%), Texas (21/315, 6.7%), Florida (17/315, 5.4%), New York (16/315, 5.1%), Pennsylvania (16/315, 5.1%), and Washington (16/315, 5.1%). We successfully enrolled participants from 80% (12/15) of the most rural states in the country.

#### Demographics

Full demographics of the study participants are reported in Table 1. The final study participants were mostly female (264/315, 83.8%), White (241/315, 76.5%), heterosexual (218/315, 69.2%), living independently (150/315, 47.6%) or with family (136/315, 43.2%), unemployed (210/315, 66.7%), and aged between 18-78 years. No demographic differences were observed following randomization between the 2 study groups.

**Table 1:** Participant demographics and clinical history at baseline (N=315).

Characteristics				Waitlist (n=161)		Active (n=154)
Age (years), mean (SD)				36.85 (10.91)		38.98 (12.30)
**Gender, n (%)**						
	Female			132 (82)		132 (85.7)
	Male			19 (11.8)		18 (11.7)
	**Transgender**			6 (3.7)		0
		MTF^a^	2 (1.2)		0	
		FTM^b^	4 (2.5)		0 (0)	
	Nonbinary			4 (2.5)		2 (1.3)
	Other			0		2 (1.3)
**Diagnosis, n (%)**						
	Bipolar disorder			61 (37.9)		50 (32.5)
	Major depressive disorder			67 (41.6)		69 (44.8)
	Schizophrenia or schizoaffective disorder			33 (20.5)		35 (22.7)
**Race, n (%)**						
	White			118 (73.3)		123 (79.9)
	Black or African American			20 (12.4)		12 (7.8)
	Asian			6 (3.7)		4 (2.6)
	Alaskan Native or American Indian			1 (0.6)		1 (0.6)
	Declined to answer			0		1 (0.6)
	More than one race			16 (9.9)		13 (8.4)
**Ethnicity, n (%)**						
	Spanish, Hispanic, or Latino			17 (10.6)		13 (8.4)
	Not Spanish, Hispanic, or Latino			143 (89.4)		139 (91.4)
**Education, n (%)**						
	Finished eighth grade			3 (1.9)		1 (0.7)
	Some high school			10 (6.2)		5 (3.3)
	High school diploma			31 (19.3)		22 (14.4)
	Some college or technical school			47 (41.6)		62 (40.5)
	Associate’s degree			15 (9.3)		21 (13.7)
	Bachelor’s degree			26 (16.1)		22 (14.4)
	Some graduate school			1 (0.6)		6 (3.9)
	Master’s degree			7 (4.3)		13 (8.5)
	Doctorate			1 (0.6)		1 (0.7)
**Employment status, n (%)**						
	Full-time			23 (14.3)		29 (19)
	Part-time			27 (16.8)		17 (11.1)
	Unemployed			105 (65.2)		105 (68.6)
**Living situation, n (%)**						
	Independent			78 (48.4)		72 (47.1)
	Living with family			71 (44.1)		65 (42.5)
	Homeless			6 (3.7)		10 (6.5)
	Substance use treatment			0		1 (0.7)
	Assisted or supported living			6 (3.7)		5 (3.3)
**Lifetime psychiatric hospitalizations, n (%)**						
	0			49 (30.4)		44 (28.8)
	1-5			69 (42.9)		72 (47.1)
	6-10			20 (12.4)		18 (11.8)
	11-15			11 (6.8)		5 (3.3)
	16-20			3 (1.9)		3 (2)
	>20			9 (5.6)		11 (7.2)
**Past-year psychiatric hospitalizations, n (%)**						
	0			127 (78.9)		114 (74.5)
	1-5			33 (20.5)		37 (24.2)
	6-10			1 (0.6)		2 (1.3)
**Frequency of auditory hallucinations, n (%)**						
	Never			82 (51.9)		75 (49)
	A few times a year			24 (15.2)		23 (15)
	Once or twice a month			17 (10.8)		15 (9.8)
	Once a week			5 (3.2)		9 (5.9)
	≥2 times a week			30 (19)		31 (20.3)
Beck Depression Inventory-II, mean (SD)				34.57 (13.34)		33.46 (13.75)
Green Paranoid Thought Scale, mean (SD)				88.24 (35.63)		86.79 (36.01)
Hamilton Program for Schizophrenia Voices, mean (SD)				19.86 (9.03)		18.42 (8.81)
Generalized Anxiety Disorder–7, mean (SD)				13.89 (5.77)		14.42 (5.16)
Sheehan Disability Scale, mean (SD)				24.11 (7.21)		24.11 (7.02)

^a^MTF: male-to-female.

^b^FTM: female-to-male.

#### Clinical Characteristics

The sample comprised individuals with bipolar disorder (111/315, 35.2%), major depressive disorder (136/315, 43.2%), and schizophrenia or schizoaffective disorder (68/315, 21.6%). Approximately a quarter (75/315, 23.8%) of the participants reported experiencing auditory hallucinations on a weekly or more frequent basis at baseline. Approximately 23.2% (73/315) of participants reported experiencing a psychiatric hospitalization in the past year, and 25.4% (80/315) of participants reported having ≥6 psychiatric hospitalizations in their lifetime. Baseline scores on clinical outcome measures were in the severe range on both the BDI-II (mean 33.95, SD 13.61) and the GPTS (mean 87.51, SD 35.79) and in the moderate range on the GAD-7 (mean 14.12, SD 5.48) and HPSVQ (mean 19.08, SD 8.89). Participants had a moderate level of disability SDS (mean 24.07, SD 7.12). Significant correlations existed between scores on the BDI-II and GAD-7 (*r*=0.69; *P*<.001), BDI-II and GPTS (*r*=0.38; *P*<.001), and GAD-7 and GPTS (*r*=0.40; *P*<.001), indicating a high degree of comorbidity within the sample. No differences were found between groups on any clinical characteristics or baseline outcome measures after randomization. Diagnosis was not found to be a significant predictor of completion at T2 or T3, with the distribution of completion resembling the distribution of participants at baseline. At T2, 20.5% (33/161) of completers had a diagnosis of schizophrenia or schizoaffective disorder, 44.1% (71/161) had a diagnosis of major depressive disorder, and 35.4% (57/161) had a diagnosis of bipolar disorder. At T3, 23.3% (27/116) of completers had a diagnosis of schizophrenia or schizoaffective disorder, 46.6% (54/116) had a diagnosis of major depressive disorder, and 30.2% (35/116) had a diagnosis of bipolar disorder.

#### Engagement Metrics

We were able to collect the CORE app use data from 51.4% (162/315) of participants. Of the 162 participants, 82 (50.6%) completed all 53 intervention levels, whereas participants, on average, completed 35 levels. Most participants reported that they would like to use CORE more often; that if they had access to CORE, they would use it; that the app was easy to use and sufficiently interactive; and that they did not need technical support to use CORE. Participants rated that they were satisfied with the CORE intervention and that they would recommend it to a friend. The distribution of participants’ responses to all usability and acceptability questions is displayed in Table 2.

**Table 2:** Participant usability and acceptability ratings (N=119).

Item	Disagree, n (%)	Neutral, n (%)	Agree, n (%)
I think that I would like to use CORE often.	8 (6.7)	26 (21.8)	85 (71.4)
I thought CORE was easy to use.	1 (0.8)	5 (4.2)	113 (95.0)
I found that the different parts of CORE work well together.	1 (0.8)	23 (19.3)	95 (79.8)
I would imagine that most people would learn to use CORE very quickly.	0 (0.0)	6 (5.0)	113 (95.0)
I felt very confident using CORE.	1 (0.8)	24 (20.2)	94 (79.0)
Overall, I am satisfied with how easy it is to use CORE.	1 (0.8)	8 (6.7)	110 (92.4)
I was able to use the *modules* quickly in CORE.	3 (2.5)	8 (6.7)	108 (90.8)
I felt comfortable using CORE.	0 (0.0)	9 (7.6)	110 (92.4)
It was easy to learn to use CORE.	0 (0.0)	7 (5.9)	111 (94.1)
Whenever I made a mistake using CORE, I could recover easily and quickly.	2 (1.7)	17 (14.3)	100 (84.0)
It was easy to find the information I needed.	5 (4.2)	16 (13.6)	97 (82.2)
The information provided for CORE was easy to understand.	2 (1.7)	10 (8.5)	105 (89.7)
How things appeared on the screen was clear.	1 (0.8)	9 (7.6)	108 (91.5)
If I have access to CORE, I will use it.	6 (5.0)	17 (14.3)	96 (80.7)
I am satisfied with CORE.	7 (5.9)	11 (9.2)	101 (84.9)
I would recommend CORE to a friend.	6 (5.0)	23 (19.3)	90 (75.6)
CORE is fun to use.	7 (5.9)	44 (37.0)	68 (57.1)
CORE works the way I want it to work.	10 (8.4)	28 (23.5)	81 (68.1)
I feel I need to have CORE.	20 (16.8)	58 (48.7)	41 (34.5)
CORE helped me manage my symptoms.	12 (10.1)	41 (34.5)	66 (55.5)
CORE was interactive enough.	6 (5.0)	26 (21.8)	87 (73.1)
I found CORE to be very complicated	108 (90.8)	7 (5.9)	4 (3.4)
I think that I would need the support of a technical person to be able to use CORE.	103 (86.6)	7 (5.9)	9 (7.6)
I thought there was too much inconsistency in CORE.	99 (83.2)	13 (10.9)	7 (5.9)
I found CORE very awkward to use.	103 (86.6)	11 (9.2)	5 (4.2)
I needed to learn a lot of things before I could get going with CORE.	105 (88.2)	8 (6.7)	6 (5.0)

### Between-Group Differences: Active Group Versus Waitlist Control Group

Analyses of differences between the active group and the waitlist control group from T1 to T2 revealed a significant treatment×time interaction effect for the BDI-II (*F*_1,313_=13.38; *P<*.001), GAD-7 (*F*_1,313_=5.87; *P*=.02), RAS (*F*_1,313_=23.42; *P<*.001), RSES (*F*_1,313_=19.28; *P<*.001), and SDS (*F*_1,313_=10.73; *P*=.001). Large effects were observed at T2 for the BDI-II (*d=*0.58), RAS (*d=*0.61), and RSES (*d=*0.64). A moderate effect was observed for the SDS (*d=*0.44) and a small effect for the GAD-7 (*d=*0.20).

**Figure 4:**
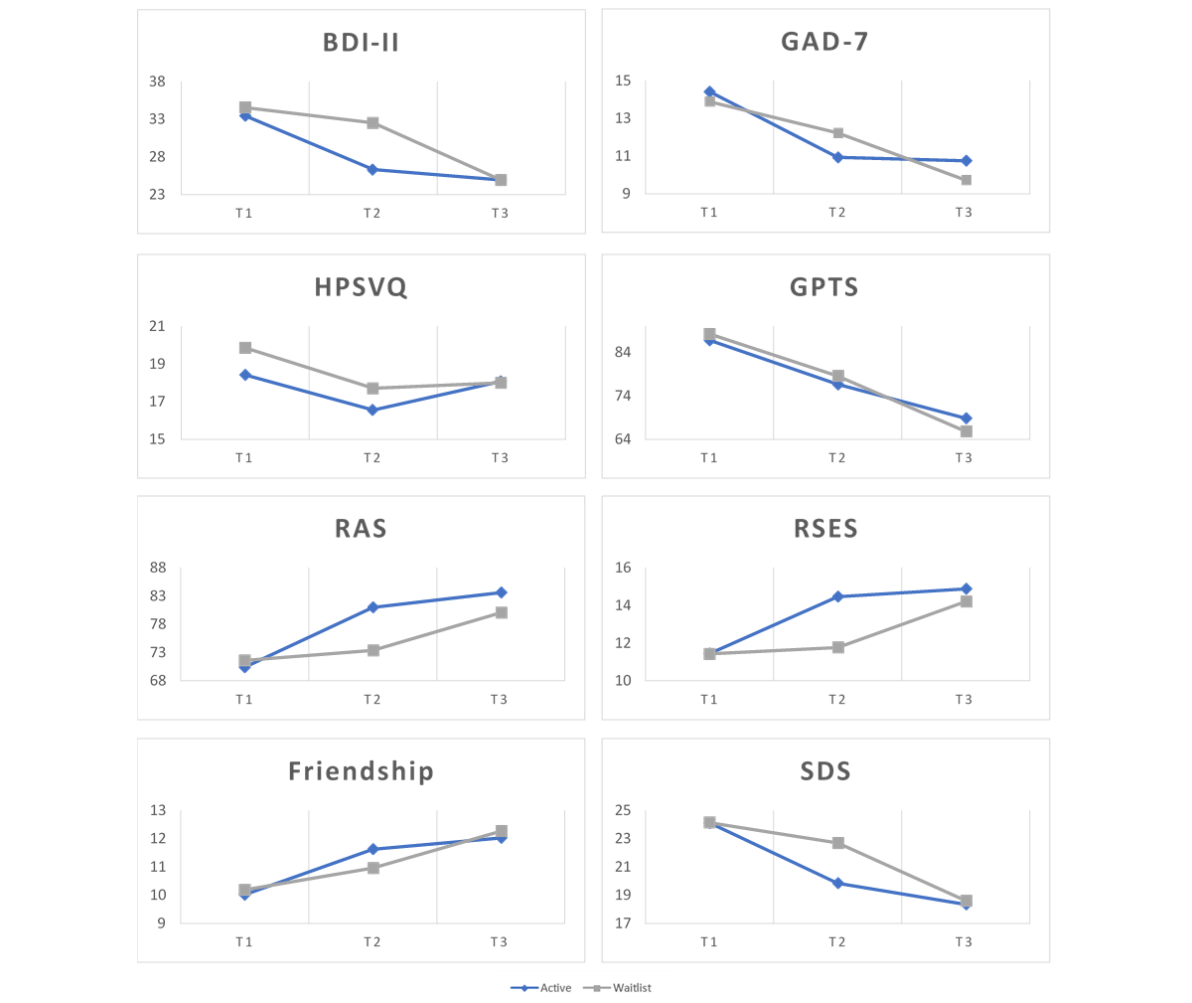
Outcome measures (y-axis) at baseline (T1), 30 days (T2), and 60 days (T3) assessments points (x-axis) for active group and waitlist control group. BDI-II: Beck Depression Inventory-Second Edition; GAD-7: Generalized Anxiety Disorder-7; GPTS: Green Paranoid Thought Scale; HPSVQ: Hamilton Program for Schizophrenia Voices; RAS: Recovery Assessment Scale; RSES: Rosenberg Self-esteem Scale; SDS: Sheehan Disability Scale.

This indicates that participants engaging in the active condition showed improvements on these outcome measures after 30 days of using the app compared with participants in the waitlist control group who did not have access to the app over the same period (Figure 4). A significant main effect of time was found for the BDI-II (*F*_1,313_=44.33; *P<*.001), GAD-7 (*F*_1,313_=66.77; *P<*.001), GPTS (*F*_1,313_=39.85; *P<*.001), RAS (*F*_1,313_=46.15; *P<*.001), RSES (*F*_1,313_=28.89; *P<*.001), Friendship Scale (*F*_1,313_=21.05; *P<*.001), and SDS (*F*_1,313_=51.644; *P<*.001). Table 3 displays the means and SDs for all measures at each time point for the active group participants and the waitlist control group participants.

**Table 3:** Comparison of outcome measures across periods for each group.

Measure		Baseline, mean (SD)	30 days, mean (SD)	60 days, mean (SD)
**Beck Depression Inventory–II**				
	Active	33.46 (13.75)	26.33 (10.43)^a,b^	24.94 (8.71)
	Waitlist	34.57 (13.34)	32.52 (11.12)	24.93 (10.59)^b^
**Generalized Anxiety Disorder-7**				
	Active	14.42 (5.16)	10.93 (4.17)^b,c^	10.75 (3.69)
	Waitlist	13.89 (5.77)	12.23 (5.00)^d^	9.72 (4.53)^b^
**Hamilton Program for Schizophrenia Voices Questionnaire**				
	Active	18.42 (8.81)	16.56 (8.54)	18.08 (6.07)
	Waitlist	19.86 (9.03)	17.71 (10.20)	18.00 (11.99)
**Green Paranoid Thoughts Scale**				
	Active	86.79 (36.01)	76.66 (23.97)^b^	68.84 (20.41)^b^
	Waitlist	88.24 (35.63)	78.59 (27.54)^b^	65.84 (24.11)^b^
**Recovery Assessment Scale**				
	Active	70.42 (16.62)	81.02 (11.01)^a,b^	83.64 (9.44)^b^
	Waitlist	71.61 (17.18)	73.38 (12.44)	80.06 (13.08)^b^
**Rosenberg Self-Esteem Scale**				
	Active	11.45 (6.39)	14.47 (4.28)^a,d^	14.89 (4.28)
	Waitlist	11.43 (5.83)	11.77 (4.96)	14.21 (5.01)^b^
**Friendship scale**				
	Active	10.02 (3.84)	11.63 (3.19)^b^	12.03 (3.00)
	Waitlist	10.19 (3.56)	10.96 (3.39)	12.27 (3.28)^b^
**Sheehan Disability Scale**				
	Active	24.12 (7.03)	19.85 (6.26)^b,e^	18.35 (6.38)^d^
	Waitlist	24.14 (7.21)	22.70 (5.83)^d^	18.61 (6.54)^b^

^a^Significance of between-group change within period (active vs waitlist control) <.001.

^b^Significance of within-group change from the previous period <.001.

^c^Significance of between-group change within period (active vs waitlist control) <.05.

^d^Significance of within-group change from the previous period <.01.

^e^Significance of between-group change within period (active vs waitlist control) <.01.

### Within-Group Change: Active Intervention Group at T2 and Maintenance at T3

A series of 1×3 repeated-measures ANOVA was used to examine intervention effects within the active group between T1 and T2 and whether these effects were maintained 30 days after discontinuation of app use (Table 3). Results of the ANOVAs showed significant within-group differences for the BDI-II (*F*_2,306_=43.59; *P<*.001), GAD-7 (*F*_2, 306_=43.71; *P<*.001), GPTS (*F*_2, 306_=34.39; *P<*.001), RAS (*F*_2, 306_=65.22; *P<*.001), RSES (*F*_2, 306_=32.85; *P<*.001), Friendship Scale (*F*_2, 306_=17.94; *P<*.001), and SDS (*F*_2, 306_=56.21; *P<*.001). Large effect sizes were observed between T1 and T2 for the active intervention group on the BDI-II (*d=*0.60), GAD-7 (*d=*0.69), RAS (*d=*0.70), and SDS (*d=*0.63). Moderate to small effect sizes occurred during this period on the RSES (*d=*0.47), GPTS (*d=*0.35), and Friendship Scale (*d=*0.39). No significant change was found at any time point for the active group for the HPSVQ (*F*_2, 306_=0.704; *P*=.51). Bonferroni post hoc comparisons revealed significant improvements in each outcome measure at T2 except for the HPSVQ. All improvements were maintained between T2 and T3, with an additional small effect noted on the GPTS (*d=*0.33), RAS (*d=*0.27), and SDS (*d=*0.24), suggesting a prolonged intervention effect.

### Within-Group Change: Waitlist Control Group at T2 and Following Crossover at T-3

Results from the active group were replicated following crossover in the waitlist control group between T2 and T3. Participants in the waitlist control group were found to have significant differences on the BDI-II (*F*_2, 322_=60.79; *P<*.001), GAD-7 (*F*_2, 322_=50.41; *P<*.001), GPTS (*F*_2, 322_=55.15; *P<*.001), RAS (*F*_2, 322_=32.03; *P<*.001), RSES (*F*_2, 322_=34.35; *P<*.001), Friendship Scale (*F*_2, 322_=26.28; *P<*.001), and SDS (*F*_2, 322_=50.93; *P<*.001). The lack of change in the HPSVQ scores after 30 days of CORE use was also replicated (*F*_2, 322_=1.14; *P*=.34). Post hoc analysis revealed that significant improvements took place for the waitlist control group between T2 and T3 for all outcome measures except HPSVQ. Effect size magnitudes were similar to those seen in the active group during the previous period, with large to moderate effects on the BDI-II (*d=*0.73), GAD-7 (*d=*0.46), GPTS (*d=*0.48), RAS (*d=*0.55), RSES (*d=*0.52), Friendship Scale (*d=*0.43), and SDS (*d=*0.67). Improvements in the waitlist control group were also found between T1 and T2 for GAD-7, GPTS, and SDS. These improvements as a result of time were sufficient to negate a between-subject effect for the GPTS but not for the BDI-II or the GAD-7. The magnitude of change observed after 30 days of CORE use between groups was equivalent across all outcome metrics except for the RAS (t_105_=2.28; *P*=.03), with the active group showing a slightly larger improvement than the waitlist control group.

## Discussion

### Principal Findings

Recent studies have demonstrated the feasibility of conducting remote trials of digital health apps for common mental health problems, including stress, anxiety, and depression [27-29]. This paper reports on the first fully remote randomized controlled trial of a smartphone intervention involving people with more severe forms of psychopathology, including schizophrenia and bipolar disorder. Building on and expanding web-based enrollment strategies used in previous works [52,53], we were able to reach, recruit, randomize, treat, and assess a sociodemographically diverse sample of participants from 45 states, far exceeding the reach of traditional localized study recruitment approaches and at a fraction of the cost. A key advantage of remote research strategies leveraging web-based recruitment advertisements lies in the flexibility they afford the investigative team to determine the geographical regions and roll out of study recruitment materials. Similar to what has been shown with other clinical populations [24], this proved to be a remarkably efficient methodology of reaching people with SMI who are more impaired and often considered more complex to engage in research [54].

This study found that a novel smartphone app, CORE, proved to be usable, acceptable, and effective in improving recovery and reducing the severity of psychiatric symptoms among individuals with SMI. Our findings align with an ample body of digital mental health research suggesting that mHealth smartphone apps can be of clinical value to people with SMI [17,55,56]. Integration of technology with human support can bolster the engagement and clinical potency of technology-based treatments for SMI [57,58]. The CORE intervention was entirely self-navigated by users in the study who received minimal or no remote technical support from our study staff. We are not advocating for a shift toward fully automated approaches as the preferred model. However, what this study did demonstrate is that when technology is designed with the characteristics of the intended users in mind (ie, in terms of functionality, accessibility, navigability, and content), deployment of specialty digital mental health tools that do not involve humans in the loop of care can also produce significant clinical benefits.

The CORE intervention builds on a digital cognitive training strategy that has been previously demonstrated to reduce dysfunctional *self-talk* and increase resilience in community-based subclinical groups [44-46]. The app included modules focused on countering maladaptive beliefs in common mental health domains (eg, self-esteem, distinguishing thoughts from feelings, social anxiety, and catastrophizing) as well as modules specifically designed for people with SMI (eg, stigma related to mental illness, threat perception and persecutory ideation, hopelessness, strengths-based recovery, self-care, and treatment seeking). Thus, it is meant to be used transdiagnostically [59].

Although this study was a fully remote trial that relied entirely on participants’ self-reports, we are confident that we were able to reach our intended target audience; most of our study sample reported having a psychiatric diagnosis of schizophrenia, schizoaffective disorder, or bipolar disorder; a history of multiple psychiatric hospitalizations; and being unemployed. Approximately one-tenth reported being currently homeless or residing in an assisted-living facility. Combined with the sample’s average baseline ratings showing moderate to severe psychiatric symptom severity and moderate to high levels of disability, our data suggest that the sample comprised people with significant functional impairment.

The waitlist-controlled study design enabled the evaluation of both between-group effects during the intervention period and within-group changes in both groups over time. The results demonstrated a link between the timing of participants’ exposure to the treatment app and when they experienced significant changes in key clinical and functional measures. A combination of factors such as simplicity, brevity, daily use, and game-like interactions may have encouraged the use of the CORE app. Daily practice comprising identification and categorization of self-statements, repeated exposure to adaptive self-statements, and psychoeducation may have facilitated retrieval of adaptive beliefs over maladaptive ones, thereby reducing the severity of symptoms [60]

This study has several limitations. The opportunity to participate was available only to individuals who used the internet (ie, to receive study recruitment advertisements) and who owned smartphones (ie, to download the CORE app) and therefore may not be representative of the full range of people with SMI. Similar to fully remote digital mental health trials involving participants with less severe psychopathology [61], our study sample was overrepresented by female participants (264/315, 83.8%), and study dropout rates were high, requiring significant data imputation. All screening and assessment questions relied on individuals’ self-reports but were not corroborated by a trained clinician or additional documentation (eg, EHR data). Replication of our findings in samples that were screened, diagnosed, and assessed by trained clinical assessors could bolster confidence in the nature of our results. Using a waitlist control is often considered a more ethical study design than offering no treatment at all or sham interventions. However, it may also have artificially inflated estimates of the intervention effects; asking participants who are ready for treatment to wait to receive CORE may have frustrated them, stalled their attempts to make independent changes, or artificially delayed their seeking other treatment options. Finally, although our measure of recovery taps *willingness to ask for help* [35,36], we did not formally evaluate changes in treatment-seeking behavior, which is an area of focus in the CORE training program and the key variable of interest.

### Conclusions

Mental health researchers, funders, industry leaders, patients, and their caregivers have been advocating for the development and deployment of effective digital health tools to improve the outcomes of people with psychiatric conditions [62,63]. The global COVID-19 pandemic has led to major disruptions in the delivery of standard mental health services and has shed light on the vulnerabilities intrinsic to complete reliance on clinic-based treatment models [64,65]. In the context of this ongoing public health crisis, new scientific evidence showing that remotely-accessed mHealth technologies such as CORE can be navigable and beneficial to people with SMI is very encouraging. Regulatory bodies have taken active steps to remove barriers to the use of digital health technologies for psychiatric disorders [66]. Currently, rapid adoption and real-world dissemination of evidence-based digital health interventions are needed if we are to shorten the science-to-service gap and help address the significant unmet mental health needs of people with SMI during the pandemic and beyond.

## Appendix

Multimedia Appendix 1 CONSORT-eHEALTH (V 1.6.1).

## Abbreviations

ANOVA: analysis of variance
BDI-II: Beck Depression Inventory-Second Edition
GAD-7: Generalized Anxiety Disorder–7
GPTS: Green Paranoid Thought Scale
HPSVQ: Hamilton Program for Schizophrenia Voices
mHealth: mobile health
RAS: Recovery Assessment Scale
RSES: Rosenberg Self-Esteem Scale
SDS: Sheehan Disability Scale
SMI: Serious Mental Illness
